# Barriers and facilitators for the implementation of blended psychotherapy for depression: A qualitative pilot study of therapists' perspective

**DOI:** 10.1016/j.invent.2018.01.002

**Published:** 2018-01-16

**Authors:** Ingrid Titzler, Karina Saruhanjan, Matthias Berking, Heleen Riper, David Daniel Ebert

**Affiliations:** a Friedrich-Alexander University Erlangen-Nürnberg, Institute of Psychology, Department of Clinical Psychology and Psychotherapy, Erlangen, Germany; b VU University Amsterdam, Faculty of Behavioral and Movement Sciences, Section of Clinical Psychology, Amsterdam, Netherlands

**Keywords:** Barriers, Facilitators, Blended therapy, Depression, Qualitative study, Therapists' view

## Abstract

**Introduction:**

Blended therapies (BT) combine face-to-face (f2f) sessions with internet- and mobile-based interventions (IMIs). However, the use of blended interventions in routine care is still rare and depends on the acceptance of key health care professionals such as the therapists. Little is yet known about the therapists' perspective on and experiences with blended approaches. The aim of this pilot study was to identify barriers and facilitators, as perceived by psychotherapists, for implementing a blended therapy for depression.

**Methods:**

Semi-structured expert interviews were conducted with five therapists, who were part of the German study arm of the FP7-project E-Compared (www.e-compared.eu). All patients (*N* = 173) were treated in the context of a registered RCT (DRKS00006866) in which the clinical and cost-effectiveness of BT for depression, consisting of ten internet- and mobile-based cognitive behavioral therapy modules and six f2f sessions, was compared to the treatment usually provided by general practitioners. To identify barriers and facilitators an interview guide based on the theoretical domains framework (TDF) was developed. The interviews were audio-recorded, transcribed verbatim and analyzed using a qualitative content analysis by two independent coders.

**Results:**

The results revealed 29 barriers and 33 facilitators, which are hindering or enabling factors on the levels of ‘implementation in the health care system’, ‘therapy’, ‘therapists’ and ‘patients’. Key barriers stated by all therapists were ‘Limited customizability and autonomy of decisions concerning blending the therapy’ (number of statements, *k* = 44); ‘Disease-related contraindications for BT’ (*k* = 25); ‘Negative affect was caused by burden through technical problems’ (*k* = 18); ‘Limited number of f2f sessions hindered the therapy process’; and ‘Establishment of therapeutic alliance was burdened by technical issues’ (each *k* = 15). Key facilitators stated by all therapists were: ‘Patients’ interest, willingness and motivation to participate’ (*k* = 22); ‘Patients' access to online content between f2f sessions and after therapy end’ (*k* = 20); ‘Preset structure of IMI-part guided the treatment course of BT’ (*k* = 18); and ‘Effective help with BT in a short time frame’ (*k* = 15), as well as ‘Reduction of the treatment gap’ (*k* = 13).

**Discussion:**

Therapists supported the implementation of BT for depression. Results indicated the consideration of a wide range of determinants: among others, the possibility of individualizing the treatment; the autonomy of decision making in respect to the ratio and number of online and f2f sessions; the necessity of providing training; the need to develop a concept of embedding BT in the health care system and funding the additional effort; and the use of sophisticated technical solutions.

## Highlights

• Blended therapies (BT) combine face to face sessions with internet- and mobile-based interventions as new treatment format
• Therapists advocated the implementation of BT for depression in routine care
• Therapists saw the possibility of combining the best of both in their daily work
• Results indicated the consideration of a wide range of hindering and facilitating determinants regarding the implementation
• BT as a 13-weeks blended approach may help to reduce the treatment gap

## 1 Introduction

Depression is the most common mental health disorder with an estimated lifetime prevalence of 12.8% (Alonso et al., 2004). Psychological treatments have shown to be effective (Cuijpers et al., 2016). However, the majority of individuals with depression remain untreated (Mack et al., 2014). Reasons for the low uptake of psychotherapy include: (a) referral rates by general practitioners to psychotherapy are often low and vary between 21% to 58% (Wang et al., 2003; Alexander and Fraser, 2008; Piek et al., 2011; Richards et al., 2004); (b) patients are confronted with long waiting lists (Kessler et al., 2001), especially in rural areas; (c) limited time resources and availability of licensed psychotherapists and; (d) attitudinal barriers often hinder patients from utilizing necessary treatments (Cuijpers et al., 2008).

Using internet- and mobile-based interventions (IMIs) to deliver psychological interventions could be a solution to overcome existing problems. IMIs have several advantages, e.g. an extensive coverage due to independence of time and space as well as therapy for patients at their own pace (Ebert et al., 2017). There is convincing evidence that such approaches are effective for depression (Ebert et al., 2015; Karyotaki et al., 2017; Königbauer et al., 2017), show higher effects with increased guidance (Richards and Richardson, 2012; Baumeister et al., 2014a) and even similar effects, when head to head compared with face-to-face (f2f) treatments (Andersson et al., 2014; Andersson et al., 2016).

However, stand-alone IMIs might not be acceptable and suitable for all patients (Ebert et al., 2015; Baumeister et al., 2014b; Baumeister et al., 2014c; Musiat et al., 2014; Apolinário-Hagen et al., 2017). Blended approaches use *“one integrated standardized CBT-treatment protocol combining face-to-face and digital components to the best clinical benefit for patients and therapists”* (Riper 2017)*.* Possible advantages of such approaches include (a) acceptability to patients for whom pure online-based interventions might not be a suitable treatment option, (b) improved treatment adherence rates compared to purely online guided interventions (Erbe et al., 2017; Klein et al., 2016), (c) the potential of time saving through processing psycho-educative content online and using therapy sessions to intensify therapy subjects or by treating more patients (Erbe et al., 2017; Sander et al., 2017; Baumeister et al., 2017; van der Vaart et al., 2014; Ly et al., 2015).

Blended therapy (BT) has not yet been well studied and its implementation is still scarce. The uptake of eMental health by routine practice is so far limited (Brownson et al., 2012; Greenhalgh et al., 2004; Kazdin and Blase, 2011). An earlier RCT compared BT for depression – f2f sessions and a smartphone application - against a full behavioral treatment and found significant improvements in both groups, but could not establish whether the BT was non-inferior to a full BA treatment (Ly et al., 2015). A recent study showed that BT for depression was more effective than psychotherapy alone and could be a promising option to consider in future treatment for depression (Berger et al., 2017). The usage of blended therapy depends on its clinical effectiveness and on its acceptance by key health care professionals. An understanding of relevant barriers and facilitators for successful implementation from the perspective of relevant stakeholders is considered to be essential (Fixsen et al., 2005). Therapists are one such important stakeholder.

Barriers and facilitators for the implementation of BT from therapists' perspective are sparsely researched. Studies indicated that the use of a blended approach in therapy has been low, with 45% of psychologists confirming use of email and mobile text messages but having a neutral attitude towards it (Wangberg et al., 2007), and with 12% of therapists confirming experience with IMIs (Whitfield and Williams, 2004). However, a promising 90% of therapists have signaled their readiness to use it in the future, especially as a supplement rather than an alternative to f2f therapy (Whitfield and Williams, 2004). To blend IMIs with f2f therapy, therapists have asked for more information as well as training to enhance usage skills (Whitfield and Williams, 2004). One of the few studies that examined attitudes towards BT for depression among care providers, found that BT was more acceptable as an effective treatment for depression for all severity levels, compared to stand-alone IMIs. However, still only 55% of care providers perceived BT as suitable for moderate depression, and 22% found it suitable for more severe cases (Topooco et al., 2017). In a Delphi study potentially suitable blended protocols (content, sequence and ratio) for depression treatment were examined by 12 therapists. The strengthening of patients' self-management was considered an important facilitator. Further key findings were that the possibility of tailoring the treatment to fit the individual patients' needs was perceived as essential, as was the possibility of adjusting the amount and ratio of online modules and f2f sessions according to the patients' problems, skills and characteristics (van der Vaart et al., 2014). A following initial pilot evaluation observed that therapists evaluated BT as a helpful tool in providing evidence-based treatment in secondary care (Kooistra et al., 2016).

A recent scoping review on determinants for a successful implementation of stand-alone IMIs for depression in regular care (Drozd et al., 2016) showed that guided support from health professionals emerged as a relevant theme. The guided support was mostly performed by therapists who aimed to enhance treatment adherence to the intervention. Important barriers that were identified included limited knowledge about and experience with these approaches, the lack of availability in routine mental health care, and a lack of training and supervision that would allow care providers to get acquainted with the treatment format. In a recent survey 88 care providers reported a moderate level of knowledge about IMIs (Topooco et al., 2017). They also expressed concerns about the limited internet literacy of some patients, concerns regarding online safety, and concerns about the potential negative effects on the therapeutic alliance, as well as on patient commitment as important barriers to implementation. Reduced cost of treatment and a better access to treatment compared to TAU were mentioned as main facilitators for an integration of stand-alone IMIs in the health care system (Topooco et al., 2017). A qualitative study with five interviewed therapists showed, that they felt hindered by the existing poor integration of a guided IMI for depression in primary care as well as the difficulties of incorporating IMIs in their work routine (Kivi et al., 2015). To enable them to use BT, therapists asked for a more flexibility in delivery of the treatment as well as the integration of f2f sessions within a blended approach.

With regard to stand-alone IMIs for the treatment of mental health disorders a literature review (Andersson et al., 2009) suggested an accurate diagnosis, a user-friendly layout, a comprehensive treatment (not overly technically advanced), and a provision for guidance by a therapist as crucial determinants for a successful implementation. Important hindering factors that have been identified for eHealth solutions in general include problems with interoperability between systems, a lack of adaptability, too little resources for familiarization with technology, poor motivation of professionals, a lack of communication about advantages (Alkhaldi et al., 2014) as well as safety concerns, a lack of confidence, and a general rejection of telecare and low operating skills (Merkel, 2017).

Despite the evidence-base for effective interventions, their transfer into routine practice is often not achieved (Grimshaw et al., 2012; Haines et al., 2004; Cunningham et al., 2010). To bridge the gap between research and practice, the use of a theoretical background regarding behaviour change in health professionals is recommended, as it guides the selection of factors that should be examined (Eccles et al., 2005; Grol et al., 2007; Michie et al., 2008). One example for implementation theories is the Normalization Process Theory (NPT), which includes factors that facilitate and prevent the routine incorporation of complex interventions into daily practice and aims to normalize their application (Murray et al., 2010; May and Finch, 2009). NPT also includes the broader organizational context, social norms and group processes, but so far NPT has been less applied in research. The ten-stages model for planning change belongs to the process models, and it aims to describe and guide the process of translating theories and research results into practice when implementing a new intervention (Grol et al., 2007; Nilsen, 2015). The Theoretical Domains Framework (TDF) aims to identify domains that influence the implementation of interventions. The TDF consists of 14 domains of potential behavioral determinants that were developed by the consensus of experts and grounded in psychological theories and that have been validated (Cane et al., 2012). It enables researchers to explain barriers and facilitators of behavior change; thus supporting the implementation of evidence-based practice. It was used in several studies for the development of qualitative and quantitative measurements (Patey et al., 2012; LA et al., 2012; Taylor et al., 2013; French et al., 2012; Amemori et al., 2011). In the current German treatment context, therapists treat their patients only with f2f therapy and do not incorporate online modules. However, blending f2f sessions with IMIs does lead to a behavior change; so the identification of influencing determinants of the therapist's behavior by the use of the TDF is crucial.

Past studies examined the use of IMIs in general in different target groups (Topooco et al., 2017; Andersson et al., 2009; Alkhaldi et al., 2014; Merkel, 2017; Andersson and Hedman, 2013). Previous studies with therapists used mainly quantitative surveys, had no link to theories of behavior change, and asked therapists, most of whom had no user experience, to report their attitude, acceptance, and intention to treat with IMIs (Wangberg et al., 2007; Whitfield and Williams, 2004; Topooco et al., 2017; Buti et al., 2013; Perle et al., 2013). One qualitative study with focus groups aimed to identify the optimal usage of blended treatment protocols for depression; but therapists differed in their user experience and assessed BT protocols that were developed on the basis of a prior quantitative survey (van der Vaart et al., 2014). A following study conducted an initial evaluation of this developed BT in specialized mental health care (Kooistra et al., 2016). This study adds to the literature several unique features: The study first examined the therapists' perspective on barriers and facilitators for the implementation of BT for depression. The study employed a qualitative theory-based approach to gain in-depth insights and addressed different domains of behavior change by interviewing the therapists after they had treated patients with BT.

The aim of this exploratory pilot study was to generate hypotheses and identify facilitators and barriers for implementing a 13-weeks BT based upon the perceptions of trained psychotherapists who experienced the application of BT in the treatment of depression while participating in the German E-COMPARED trial.

## 2 Methods

### 2.1 Design of the trial

The current research project was part of the German study arm of the European Project E-COMPARED (www.e-compared.eu), that was the first multi-center study which aims to evaluate the clinical and cost-effectiveness of a blended depression treatment across eight European countries (Kleiboer et al., 2016). The main objective in Germany was to evaluate the effectiveness of blended Cognitive Behavioral Therapy (bCBT) for adults with a diagnosis of Major Depressive Disorder (MDD) compared to treatment as usual (TAU) in primary care. BT was conducted by psychologists in a publically funded outpatient university clinic. TAU was the routine care patients receive by GP, the most common treatment for depression in Germany. Patients were recruited through local general practices from February 2015 to August 2016 and randomly assigned to the treatment conditions TAU (*n* = 87) vs. bCBT (*n* = 86).

The study was approved by the ethics committee of the German Society of Psychology (DGPs) and registered in the German Clinical Trials Register (DRKS00006866, on 2 December 2014). All therapists provided informed consent.

### 2.2 Blended therapy

The blended approach is a new treatment format in Germany and in other countries. It has an innovative character (Kemmeren et al., 2016) regarding the inclusion of web- and app-based components and ecological momentary assessment. BT for depression consisted of six therapy modules on an online platform (Moodbuster), a mobile app, as well as six f2f sessions with a therapist. It was delivered within 10 to 13 weeks by psychologists as an alternative treatment for general practitioners' care. This duration of treatment is considered as short time intervention in the German health care system. The platform provided access to treatment modules, homework exercises, mood graphs, a calendar, and a messaging system. A therapist portal enabled therapists to monitor their patients' treatment course, provide feedback on exercises and progress or write reminder or motivation emails to enhance adherence. The online modules based on established CBT-principles for MDD and consisted of 10 lessons for psychoeducation, behavioral activation, cognitive restructuring, problem solving, sports and exercises, relapse prevention. After editing two mandatory online lessons, patients had access to all of the remaining modules except the relapse prevention module. That module was released only after finishing their last f2f session. One module was optional. The patients were instructed to work on one module lesson at a time and to complete it in one week. Together with his or her therapist, each patient decided upon the sequence in which to complete the modules. Patients worked on the online lessons weekly at home, used the app for daily ecological momentary assessments (mood state, activities, sleep habits), and got automated tailored reminders and individualized motivational messages. On-site f2f therapy started with a technical introductory, lasted 50 min every two weeks, and enabled therapists to refer back and forward to web-based modules and mobile-based ratings in order to consolidate themes and structure the treatment.

The patients treated by the interviewed therapists of this study (*N* = 71) were on average 43.4 (*SD* = 13.16, Range = 19 to 70) years old, mostly female (59.2%), highly educated (53.5%), and had a paid job (71.8%). 71.8% had no earlier experience with psychotherapy. 90% of patients completed all six f2f sessions, 75% all ten online sessions. They had 5.7 f2f sessions on average (SD = 1.06, Range: 1 to 6) that lasted 57.3 (SD = 5.02, Range = 48 to 77) minutes.

### 2.3 Participants

Eight therapists treated patients in the study and were invited to join expert interviews. In total, five therapists took part in semi-structured interviews. Three could not participate because of interference with current occupation, as they no longer worked for the trial center.

All participants were women, young (*M* = 28.40 years old, *SD* = 2.52), held university degree in psychology (master, diploma) and were undergoing further training as advanced clinical CBT-therapists. Their average experience as therapist was 24 months (*SD* = 29.39), but without previous experience in BT (see Table 1). They were employed between May 2015 and December 2016 and treated on average 16 patients with BT for depression. All the therapists were trained in the BT protocol under the supervision of an experienced, licensed psychotherapist.

**Table 1:** Sample characteristics of expert interview participants.

Sample characteristics of expert interview participants.	
Characteristic of therapists	Sample (n = 5)
Age (years): *M* (*SD*)	28.40 (2.60)
Female gender: *n* (%)	5 (100)
Education level - university degree: *n* (%)	5 (100)
Duration in training as CBT therapist (months): *M* (*SD*)	19.00 (11.71)
Experience as a therapist (months): *M* (*SD*)	24.00 (29.39)
Duration of employment as blended therapist (months): *M* (*SD*)	9.40 (1.67)
Number of patients treated with bCBT: *M* (*SD*)	16.40 (2.30)

### 2.4 Design of qualitative study and data collection procedures

We developed a qualitative design method with a theory-based approach to gain insights into the experiences of the therapists who worked with BT. This method is recommended as the best approach to identify barriers and facilitators (Campbell, 2000; Campbell et al., 2007) and to bridge the gap between research and practice (Huijg et al., 2014).

The interview guide was developed after a literature review according to the theoretical domains framework (TDF) with 14 domains of potential behavioral determinants (Cane et al., 2012) and scholarly exchange with clinical experts. The semi-structured interview guide (see Table 2) should elicit therapists' perceptions of barriers and facilitators while applying BT. When needed, guided prompts enabled gathering more information.

**Table 2:** Theoretical domains framework (Cane et al., 2012) and exemplary questions of the interview guide.

TDF - domains	Interview questions
D1 knowledge	What do you know about blended therapy and its effectiveness?
D2 skills	Which skills and competences do you consider as necessary to treat patients with blended therapy?
D3 social/professional role and identity	Do you see treating patients with blended therapy as part of your role?
D4 beliefs about capabilities	How difficult or easy is it to treat patients with blended therapy?
D5 optimism	Based on your experience, how confident are you that the use of blended therapy will run optimally?
D6 beliefs about consequences	What do you think about the benefit of blended therapy for your patients?
D7 reinforcement	To which amount are the benefits of blended therapy for patients sufficient to justify the treatment via blended therapy?
D8 intentions	How much of a priority is blended therapy in the care of patients with depression?
D9 goals	How do you feel about the goal to implement blended therapy into the health care system in a way that you could use it in your future professional life?
D10 memory, attention and decision processes	To what extent can you imagine that blended therapy for depression will be something you usually do or remember to do in the future?
D11 environmental context and resources	How could blended therapy serve as facilitator during your work as a therapist and in simplifying administrative tasks?
D12 social influences	Did colleagues or patients/relatives ever prompt or encourage you in the treatment with blended therapy?
D13 emotion	When you think about using blended therapy what kind of feelings emerge?
D14 behavioral regulation	Are there procedures or ways of working that encourage treatment via blended therapy?

All semi-structured interviews were conducted by the first author (IT) at the workplace of the interviewees between April and November 2016. The average duration of an interview was 99 min (*SD* = 12.10). Interviews were audio-recorded and then transcribed by the second author (KS) verbatim based on a transcription guide. Anonymity was assured by using code numbers instead of names.

### 2.5 Data analysis

A qualitative content analysis was conducted, drawing on inductive and deductive approaches and using standardized methodical steps in qualitative research (Mayring, 2010; Mayring, 2002).

Following the inductive approach, codes were developed from the raw data, based on themes relevant to addressing the research question. First, a list of codes based on 60% of material was developed after identifying emerging (sub-) themes and discussing excerpts, the organization of the categories, and the possible meanings of text frames within consensus meetings (KS, IT). Second, the entire interview material was read, labels were attached to text parts related to the list of codes, and new emerging themes were added to the list of codes. During this coding process, new categories emerged and old ones were changed in order to fit the data. A sentence or paragraph could be coded as containing aspects from one or more categories. A preliminary categorization guide based upon 100% of the material was developed and discussed in consensus meetings. The consensus meetings reached final agreement on code definitions and excerpts, the structure of the code system, a final list of codes, and coding rules. The clinical expertise of a CBT therapist (IT) enriched the study's data interpretation by discussing the possible meanings of text frames in the context of applied CBT. Third, two coders (KS, SH) independently coded all transcripts of the interviews with the five therapists in accordance with the final code list. There was sufficiently high agreement between the coders with Cohen's Kappa k = .85 (Wentzel et al., 2016). Forth, data saturation (i.e., each category being mentioned by at least two therapists) was not expected for the small sample size of this exploratory study. However, 97% of the categories were mentioned by at least two or more therapists, while only two of the categories were mentioned by only one therapist. This level of data saturation is valid for case analysis (Glasgow et al., 2003). Fifth, to ensure the validity of the identified categories, the five therapists were questioned as to their individual agreement with the resulting barriers and facilitators as indicated by data analysis. The agreement rate per individual therapist was *M* = 90% (*min* = 78%, *max* = 100%). In total, all five of the therapists agreed with 66% of the themes, while four out of five therapists agreed with 84% of the themes. Thus, the identified barriers and facilitators yielded a good validation result with an agreement rate of at least 40% to 100%. Sixth, key themes were defined as emerging themes mentioned by all five therapists. These key themes are reported in the results section. All categories, including other themes mentioned by four or less therapists, are listed in tables and figures.

Following the deductive approach, coding of the whole material was done by referring emerging themes to the domains/constructs of the TDF and classifying the reported experiences within each domain as barrier and facilitator. The independent coding by two persons yielded a mild to moderate agreement with Cohen's Kappa κ = .68 (Brown and Hauenstein, 2005).

All qualitative analyses were conducted with the software tool MAXQDA. Consolidated criteria for reporting (COREQ) were followed (see Supplementary Table 1).

## 3 Results

In sum, 29 barriers and 33 facilitators were identified. Each barrier and facilitator was categorized and abstracted into one of four main areas, as follows: (a) hindering and enabling factors that can be useful to develop an implementation strategy that embeds a 13-week BT to treat depression in Germany's primary care system, while considering the existing framework, were classified under the topic ‘implementation in the health care system’ (see Table 3); (b) hindering and enabling factors that influence the therapy process and the interaction between therapist and patient were labeled as ‘therapeutic factors’ (Table 4); (c) hindering and enabling factors that personally influenced the therapists‘ behaviour, cognition, and feelings were clustered under the label ‘therapist factors’ (Table 5); (d) while, likewise, hindering and enabling factors that personally influenced the patients' behavior, cognition, and feelings were clustered under the label ‘patient factors' (Table 6).

**Table 3:** Identified facilitators and barriers for blended therapy from therapists' perspective (*N* = 5): Sub-categories on the level of ‘Implementation in the health care system’ with definitions and supporting quotations.

Categories	Therapists			Definition	Supporting quotations
	*N*	%	*K* no. of excerpts		
Facilitators (*n* = 15)			152		
Press and publicity work to enhance prominence and acceptability	5	100	13	Disseminating activities for the professionals and population (e.g. providing information, advertisement) enhance prominence and acceptability and increase a functional sociopolitical environment	*“Health care workers, GPs and consulted doctors must know, that BT is provided.”* (*T008*)
Reduction of the treatment gap	5	100	13	Overcoming the treatment gap through an additional treatment option, compensating low resources in therapists and enabling treatment start to an earlier point of time	*“In the end it is possible to provide more therapy. Since BT is also something young therapists and therapists in training can easily work with, certainly more therapies could be provided.” (T011)*
Intuitive usability and logical structure of online-platform	5	100	13	The online-platform has a logical structure and high usability, the module order is clear.	*“I think it is pretty good right now. Usability (referring to understandability and intuitivism of and simplicity the platform and processes) is a very important issue that has to be considered and provided.” (T004)*
Modern and contemporary treatment approach	5	100	12	BT by using online components is modern, contemporary and easy to implement since nearly everyone has daily access to the internet	*“Everyone thought it was a cool and useful project. So, I think it was really contemporary.”(T007)*
Adequate treatment approach for depression	5	100	20	Treatment approach for recurrent or first-time unipolar mild/moderate depressive episode, dysthymia, exclusionary depressive symptoms. Online content is therapeutic useful and relevant.	*“I really liked the nice website with all the really, really well-prepared lectures, information, examples and exercises.” (T011)*
Education and training offers	5	100	10	Introduction workshops, manuals, test-accounts for therapists, practicing in role plays, feedback reports of patients as useful parts of training offers	*“After five years there are update-supervisions or trainings, where therapists can get a new certificate. This way you are also staying in contact with the founders.” (T004)*
Accessibility to a new group of patients	5	100	7	Attractive approach for hardly reached patient groups (e.g. reservations of traditional therapy, men, young patients)	*“I think you might reach more or different patients.” (T008).*
Low-threshold treatment	4	80	14	Easy accessibility and a low threshold approach for a first-time intervention	*“Definitely the low-threshold treatment and the easy accessibility for patients. Also the fast inclusion.” (T007)*
Prevention approach for subclinical symptoms	4	80	9	Good approach for patients with minor depression and subclinical symptoms who would usually not get any treatment	*“I would definitely use it for prevention.” (T007)*
Availability of IMIs on an online platform with access to updates	4	80	6	Provider offering an online platform with a package of different online modules with continuous updates and improvements to use in BT	*“I think it is good, if you are able to buy a package like you would buy psychological tests. So, people know how much they pay and can then be sure a team cares about the contents and is constantly updating it. That would be perfect!” (T010)*
Ensuring widespread dissemination and availability of BT	3	60	9	Widespread dissemination, accessibility and supply on many different levels and for all patients	*“It is then offered broadly. Not only some patients are able to get it.” (T011)*
High treatment quality through guideline- and evidence-based IMIs	3	60	9	Ensuring a high treatment quality of the IMI-part through constant evaluation by research institutes, the use of empirical proved manual-based methods and orientation on guideline recommendations	*“There are some basics that I want my patients to have. The possibility to have a high quality treatment, would be the biggest advantage for me. I can control effects and benefits of the treatment by providing some good contents to everyone in some way. So, patients certainly get the contents that have the most empirical evidence.”* (T007)
Treatment approach for relapse prevention	3	60	7	Suitable treatment approach in aftercare or as a relapse prevention	*“Overcoming the treatment gap or as a relapse prevention after a hospital stay. I think it is especially important to patients having less structured therapy after a hospital stay. Having additional support.” (T007)*
Service: Technical support	3	60	6	Desire for good technical support and help with all technical questions	*“Who is responsible for all technical issues and who can patients ask for help? How can we make technical support as easy as possible for patients?” (T010).*
Preventing symptom deterioration and inpatient care	1	20	4	Symptom deterioration or inpatient care could be prevented through a 13-weeks treatment while waiting for therapy	*“It is also about averting deterioration to avoid more intensive care or at least hospitalization.” (T007)*

Barriers (*n* = 6)			50		
No funding solution for online-services and additional therapeutic effort	4	80	12	No existing funding concept in the health care system, uncertainty about possibilities to reimburse	*“It depends, of course. If you have to buy it somehow for around 1000€, it's not that preferable.”* (T008)
Immature and problematic technology	4	80	12	Slow internet connection, faulty interface, buttons only in English	*“In my opinion there are still technical problems in development. Sometimes buttons are not displayed at all or in English while they should be in German.”* (T004)
Not part of regular care	4	80	8	Not part of regular care with free provision of all BT components	*“If it is provided and paid by health insurances.” (T008).*
Embedding into health care system unclear	4	80	8	Unclear concept for embedding BT into routine care	*“Although, we'll see how it will actually work: How people can use it, how the system is organized, if GPs are joining or if it is maybe more part of a daycare clinic or something like that. I'm a little skeptic, how this will actually work.”* (T011)
Immature aspects of the online modules	3	60	7	Some immature aspects at the online-platform need improvement, e.g. no connection between calendar and smartphone	*“The modules ‘Psychoeducation’, ‘increasing behavioral activation’ and the calendar could be easier. Especially the calendar could be more practical. Some clients suggest to connect it with google. You could maybe really think about that with technical development.”* (T010)
Limited data safety	3	60	3	*Faulty or missing data safety decrease motivation to work with BT*	*“And of course there are many fears coming up with this. We are often talking about data safety, encryption,* etc. *It should not be mentioned in the same way as „surveillance society.”* (T011)

**Table 4:** Identified facilitators and barriers for blended therapy from therapists' perspective (*N* = 5): Sub-categories on the level of ‘Therapeutic factors’ with definitions and supporting quotations.

Categories	Therapists			Definition	Supporting quotations
	*N*	%	*K* no. of excerpts		
Facilitators (*n* = 7)			94		
Patients' access to online content between f2f sessions and after therapy end	5	100	20	Possibility to access information and exercises online without therapist presence between f2f sessions and after the end of treatment patients can repeat modules	*“Patients can revise what we talked about, get access to all exercise and add something later on. Furthermore, they can repeat some modules after the official end of therapy.” (T007)*
Preset structure of IMI-part guides the treatment course of BT	5	100	18	Guidance and support for patients and therapists via online platform. Structured treatment course and leading path through online modules.	*“I think it is the simple fact that contents are written down there. You can see and use the platform. You have to learn how to use it first, of course, but it is a preset structure you can use.” (T010)*
Effective help with BT in a short time frame	5	100	15	Benefit of therapy and decline of symptoms in a short time	*“Just when you came and everything was bad, there was this huge benefit in such a short time.” (T011)*
Therapists' online monitoring of treatment course and assessments	5	100	10	Online components allow assessment and monitoring of the therapy process and facilitate the preparation of f2f sessions	*“I can see the results before the next session. I know the patient worked on this at home and also when and how long it took him/her to do this.” (T010)*
IMIs as useful complement to f2f sessions	4	80	11	High acceptance of online lectures as additional offer to pure f2f therapy	*“I already said that I like it as an addition. (..) On the one hand as a supplement to usual f2f therapy or as a combined therapy with five or more f2f sessions.* “*(T008)*
Personal contact as a key factor of therapy	4	80	10	Personal contact as a key factor for therapeutic alliance, motivation to work and ability to interact with patients.	*“I think, personal contact will be rated as very important. It is more difficult to motivate people without a therapeutic relationship.”(T007)*
Strengthening of patients' self-efficacy and self-management skills	3	60	10	The self-help parts of BT and an earlier experience of therapy profit strengthen the self-efficacy and self-management skills	*“There is a stronger emphasis on self-efficacy, which represents a professional treatment.” (T007)*

Barriers (*n* = 8)			111		
Limited customizability and autonomy of decisions concerning blending the therapy	5	100	44	Lack of flexible customizability and individualization due to strict guideline rules for using BT. Wish of more autonomy of decision concerning number and ratio of f2f and online sessions, number of treated patients, application and integration of online modules.	*“Another partly difficult thing was that the modules were generalized. It was hardly possible to give a module about activity to one patient and a module about ‚saying no‘to another one. However it was already obvious that the ‚one size fits all‘approach is not working everywhere.* “(T007)
Negative affect was caused by burden through technical problems	5	100	18	Patients as well as therapists experience frustration, demotivation and anger caused by frequent, unsolved technical problems.	*“Technical problems annoyed me at some time point. There were always some kind of technical issues unsolved and I can't help and had to contact the IT.”* (T008)
Limited number of f2f sessions hinder the therapy process	5	100	15	Too few f2f-sessions decrease the benefit of the intervention due to e.g. few possibilities of stabilized changes	*“Moreover, for 90% of my clients six sessions were too few.”* (T004)
Establishment of therapeutic alliance was burdened by technical issues	5	100	15	Therapeutic alliance is burdened through reminding mails and the technical explanation module as starting point of the therapy	*“I think it is difficult for clients who have already problems with building up relationships.”* (T008).
Limitation of depression treatment	3	60	6	No offer of online modules for different mental disorders or comorbidities that were necessary	*“This module was only for depression, so it was limited anyway and other disorders of this spectrum were missing.”* (T011).
Distraction from therapeutical aims caused by technical problems	3	60	5	Searching for solutions for technical problems takes time which shortens therapy time and distracts the therapy process	*“The module didn't work for a long time with one of my patients. So, we mainly talked about technical problems. That was a big distraction.”* (T011).
Expectation of a non-stop availability of the therapist	3	60	4	Difficulties for patients and therapists to find clear boundaries. False expectations of an immediate communication option.	*“Maybe if patients think, they have a direct access to the therapist, and try to use this in a certain situation for calling for help (..) However, we are very clear about not sitting in front our computers 24/7.”* (T010).
Restrictive possibilities for coping with acute crisis	1	20	4	Lack of nonverbal input and therefore difficulties to recognize crises early and react accordingly in a 13-weeks BT without weekly f2f sessions	*“I had negative experiences. When I was told by the online platform, that someone was in a crisis, I felt a little bit powerless. First because working in home office, I didn't have a chance to call or get in contact directly. So, I had to do it online and with a delay sometimes.”* (T007).

**Table 5:** Identified facilitators and barriers for blended therapy from therapists' perspective (*N* = 5): Sub-categories on the level of ‘Therapist factors’ with definitions and supporting quotations.

Categories	Therapists			Definition	Supporting quotations
	*N*	%	*K* no. of excerpts		
Facilitators (*n* = 8)			58		
Time savings in therapy	5	100	13	Facilitation of therapeutic work and saving time by pre-gathering some information online	*“It will be easier, if two therapists have to share rooms, too. Because at the internet therapy patients only show up every second week and so you have more resources. I experienced that: If our patients had been here every week, it would not have worked with our room capacity, but like this it was fine.” (T011)*
Useful digital tool kit	5	100	9	Easement of therapeutic working process through provision of online input, accessible repertoire of information and online exercises; therefore the therapist is being able to focus on some specific issues in therapy by using digital tools	*“It is helpful to know that you have this repertoire of exercises and you can focus on one or the other with every client. Maybe there are some lections that are less useful for some of them and that is also okay.” (T010)*
Awareness of the role and tasks as a BT-therapist	5	100	8	Awareness of the role and tasks of a BT-therapist and knowledge about the integration of web- and app-based parts in f2f sessions as well as knowledge about using websites are helpful aspects. Therapists reported sufficient knowledge and skills and no identity conflict with their role.	*“HOW can I now integrate this web-based or app-based parts in my therapy session? This is an important issue in my opinion.” (T011)*
Beneficial therapeutical skills	5	100	8	Empathic attitude, frustration tolerance, persistency, open-mindedness, enthusiasm for online-therapy, good writing skills as well as building up a therapeutic alliance in a short period of time are important	*“I also think you should have a certain enthusiasm for this. Not only as a skill, but as a requirement. I have to be able to motivate people and show them why additional working with online modules is worth it.” (T007)*
Positive attitude	4	80	8	Therapists reported a positive attitude towards implementing BT. They were optimistic according to a positive course and outcome.	*“I'm definitely a fan of this so called combined treatment.” (T007).*
Technical knowledge	4	80	5	Existence of basic technical knowledge of e.g. functions of a computer, smartphone or apps	*“I think I certainly need a little bit more technical knowledge and skills than usual, so I can for example answer basic questions. This is also showing some competence.” (T007)*
Varied activity that compensates for demanding f2f sessions	3	60	4	Variance in therapeutic work through e.g. organizational tasks or writing online feedbacks. It brings pleasure and a compensation for demanding f2f sessions. Therapists like it.	*“I like this work. I'm not only sitting here, preparing therapy and sitting here again, but also working on the computer, organizing the process* etc. *It is a pleasant work in my opinion. So, for ME it would be good to work in this setting.” (T011)*
Having worked with BT increases intention to use it	2	40	3	Working experience with BT increases the motivation and intention of therapists to use it again in the future. They found it easy to draw attention on it.	*“Since I already worked with this now, I think I'll keep in touch and updated with all the apps and developments that will come. (T011)*

Barriers (n = 7)			50		
Negative effects and time burden caused by reminder mails	5	100	7	Negative perception of need to remind patients to work on their online modules. Therapists found it frustrating, onerous and time-consuming.	*“Reminding people takes most of the time. ‘Do this, do that ‘(..) It is a little exhausting and sometimes frustrating.”* (T010)
Additional organizational effort	4	80	18	Therapists must find additional time for online based work e.g. checking the online platform, writing feedbacks, solving technical problems and controlling the processes. This leads to an ambivalent perception of the cost–benefit ratio and reinforcement.	*“It's an organizational effort. Even if I don't see the patient and work with him/her directly, but work ‚for him/her’ at the online platform, I must take my time for that. That means the therapeutic effort is the same, even if it is online therapy, which is important to remember.”* (T004)
Time pressure	3	60	8	Too less time to treat all issues properly and too much input for one session cause stress and time pressure	*“In the beginning knowing that there are only 6 sessions, pressured me a lot. I thought I have to do my very best in those sessions and use them perfectly.”* (T010)
Fear of negative consequences for own professional group	3	60	6	Fear of reduced therapeutic resources through decreasing costs and jobs losses, fear of replacing or shortening therapy	*“Oh now, we can hire less therapists or they can take more clients, since they have more time. Even if there is a HUGE demand of usual psychotherapy and MORE therapists.”* (T011)
Skeptical attitude of other therapists expected	3	60	4	Therapists will continue with usual psychotherapy, because of more knowledge and more security	*“I'm not sure if therapists in general will adopt and supply it, if we make it possible. And if not, the lack stays.”* (T011)
Fear of negative consequences for patient care	2	40	4	Fear of worse patient care by replacing traditional therapy with online therapy	*“Of course, a first reaction could be: ‘You don't want me to come here too often, since you are sending me away with some pseudo-internet-help.’”* (T010).
Deceleration of routines caused by asynchronous communication	2	40	3	Decreased quality of communication due to asynchronicity and less possibilities of sorting things out, thus routines are decelerated	*“Sometimes I rather wanted to call the patient and sort things out quickly than writing a message. That's okay, if you cannot reach the patient at all* via *mail. But anyway, I think sometimes it'll ruin normal routine because you must write a message, then the patient must respond, then you have to write again and so on.”* (T011)

**Table 6:** Identified facilitators and barriers for blended therapy from therapists' perspective (*N* = 5): Sub-categories on the level of ‘Patient factors’ with definitions and supporting quotations.

Categories	Therapists			Definition	Supporting quotations
	*N*	%	*K* no. of excerpts		
Facilitators (*n* = 3)			40		
Interest, willingness and motivation to participate	5	100	22	Patients are interested, show positive attitude and motivation for this innovative and exciting therapy	*“None would have EVER drawn so many mood curves with a pen. But there is a high intensity of drawing those in the app. I think this is useful even after the end of the project.” (T007)*
Location and time independence	4	80	13	Location and time independence while working with therapeutic contents online. The IMI part and fewer f2f sessions of BT enables an adaption to personal life circumstances, e.g. job.	*“You can consider life circumstances. If someone has to drive long ways to work or is traveling a lot for work or working in shifts and being not able to participate f2f sessions regularly, you can say working online is perfect. Flexibility and adaptively to any life circumstances.” (T007)*
Possibility to work at own pace	4	80	5	The IMI part of BT enables patients to decide when and how they intensify their therapy process	*“Patients have enough time at home to think about the online content, to repeat some exercises or modules.’ (T008)*

Barriers (*n* = 8)			96		
Disease-related contraindications of BT	5	100	25	Characteristics of disease are inadequate for BT (e.g. level of severity, lack of energy, lability, suicidality)	*“I think it is difficult for example with chronical depression or the latest of many recurrent depressive episodes. Especially because of the short time.”* (T008)
Reservations and less engagement in IMI-part	5	100	13	Less acceptance of IMI-part or perception as temporary intervention to bridge the time until start of traditional therapy or perceiving online components as less important than f2f therapy	*“In the end, I think some of them did not think of those exercises as an important part of the intervention.” (T011)*
Poor completion of online components	4	80	16	Superficial, careless completion or skipping of the online modules	*“There is probably a third group: Patients who could benefit but don't care enough and work hard enough.”* (T011)
Overburden through overload	4	80	15	Overburdened patients due to high amounts of input, too fast completion, too complex modules	*“It was deterrent for some patients. There are seven steps. What if step one feels complex already? What happens next?”* (T008)
Low technical affinity	3	60	8	Insufficient affinity for technology, little experience with computers, annoyance from working with computers	*“You can definitely see from the beginning on, that using different websites is not that easy for everyone. The moodbuster website is very simple, but if you don't use a computer or the internet that often, it is still hard.”* (T011)
Elderly patients are perceived as unsuitable	3	60	7	Inadequacy of online therapy for elderly patients	“*I cannot really picture elderly people working with it. I think we had some of them, (..) but still.”* (T008)
Cognitive impairment/lack of structuredness/visual impairment	3	60	6	Cognitive impairment, lack of structuredness or visual impairment hinder use of BT	*“For patients who have problems with structuredness that are not related to depression, it is hard to sit down and work on the program.”* (T004)
Low added value for patients with therapy experience	2	40	6	Low additional value and less motivation for patients knowing the contents already through previous therapy	*“You have to consider the previous therapy experience of your patients. I'm not sure if it helps people who have been in therapy for years and already know basic exercises backwards.”* (T010)

All barriers and facilitators are described alongside a definition and supporting quotation. In total, the most facilitators (*n* = 15) were found on the level of ‘Implementation in the health care system’, whereas the most barriers (each *n* = 8) could be identified on the level of ‘therapeutic factors’ and ‘patient factors’.

### 3.1 Barriers

**Fig. 1:**
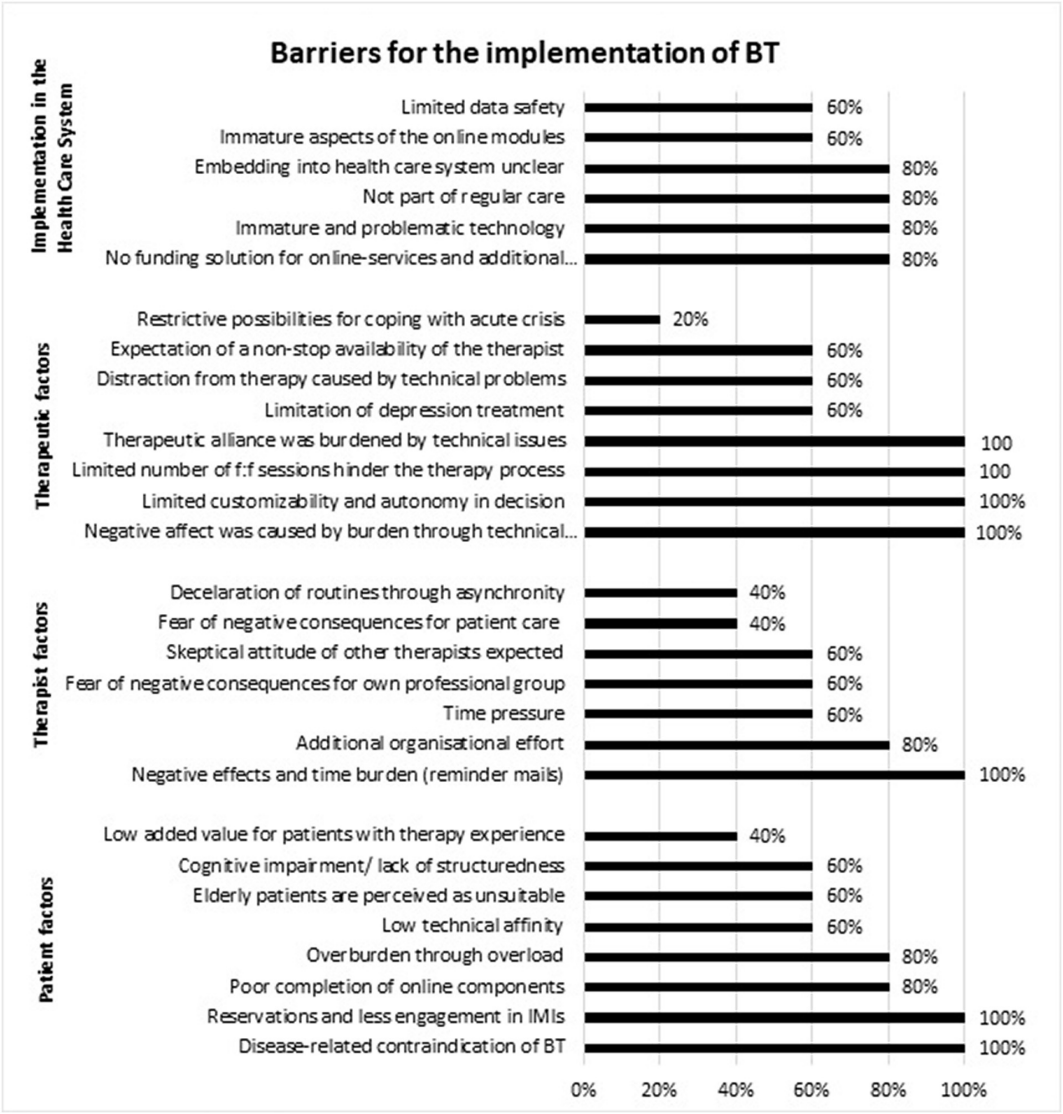
Barriers per main area and the percentage of therapists mentioning it.

In total, therapists perceived more barriers regarding BT on the level of ‘patient factors’ and ‘therapeutic factors’ compared to ‘implementation in the health care system’ and ‘therapist factors’. 24% (*n* = 7) of the identified 29 barriers were mentioned by all therapists, and 48% (*n* = 14) by at least four of the five therapists. Below, key barriers mentioned by all five therapists are described with a quotation illustrating the therapists' view. All categories and subthemes and the number of therapists who mentioned the respective barrier are listed in Fig. 1.

#### 3.1.1 Implementation in the health care system

In this category none of the barriers were reported by all of the therapists, therefore barriers that were mentioned by four therapists are described below.

Four therapists mentioned the immature technology and other technical problems, such as a slow internet connection or faulty interfaces, as important hindering factors. Furthermore, they noted that the therapist should have some additional resources to provide BT, such as access to the platform, the possibility of checking online activities, the capability to write and send reminders and motivational emails to their patients, and training sufficient to enable the therapist to solve minor technical problems. The lack of a concept to fund these additional activities was perceived as a barrier. But also the lack of a concept by which to embed BT into the health care system was described as main barrier, because its currently not part of routine care.

*“Although, we'll need to see how it will actually work: How would patients use it; what structures should be given? Would it be linked to a therapeutic private practice or included in outpatient mental health clinics or something? I'm a little skeptical; what will it look like actually.” (T011).*

#### 3.1.2 Therapeutic factors

All therapists criticized the ‘one size fits all` approach, the standardized treatment procedure for depression, and the limited possibilities to tailor the treatment to the patients’ individual needs.

*“Another partly difficult thing was that the modules were generalized. It was hardly possible to give a module about activity to one and a module about ‘saying no’ to another patient. However, it was already obvious that the ‘one size fits all’ approach is not working everywhere.” (T007).*

Consistent with this perception, all the therapists wished to be autonomous in deciding how to use online lessons in their patients' therapeutic treatment. The therapists wanted to choose the number and ratio of applied f2f and online sessions, to choose the content, and to decide the sequence of online modules and which patients they treat with BT. The therapists recommended 11 (*min* = 7, *max* = 25) f2f sessions on average for patients in the short time blended approach in Germany. All wanted more flexibility and no pre-described protocol regarding the ratio of online and f2f sessions, as the following examples show:

*“A certain kind of flexibility. For example with the number of f2f sessions. So no one should say that with using this therapy there are only 6 f2f sessions possible, but more the possibility to decrease or increase this number.” (T010).*

All therapists further mentioned that the low and limited number of f2f sessions for each patient reduced the potential benefit of the approach. For example, the time was not sufficient to help patients to stabilize achieved therapeutic gains.

*“Moreover, for 90% of all my patients six sessions were too few.” (T004).*

Moreover, permanent or unresolvable technical problems caused anger, frustration, and demotivation in both patients and therapists. The therapists all agreed that these barriers and providing technical introduction in the first therapy session hindered the establishment of the therapeutic alliance.

#### 3.1.3 Therapist factors

There was only one barrier stated by all therapists. They perceived the need to remind patients to complete online-lessons as frustrating, onerous, and time-consuming, as one therapist outlined:

*“Reminding people takes most of the time. ‘Do this, do that’ (...). It is a little exhausting and sometimes frustrating.” (T010).*

#### 3.1.4 Patient factors

Regarding important factors on the patient level, all therapists assumed that those patients having reservations towards IMIs may not be suitable for this treatment approach and should therefore have the possibility to choose stand-alone f2f treatment. All therapists reported the impression that some patients perceived BT as a temporary intervention to bridge the time until their start of a traditional f2f therapy, others perceived online components as less important than f2f therapy.

*“In the end, I think some of them did not think of those exercises as an important part of the intervention.” (T011).*

In addition, they pointed out potential disease-related contraindications for the use of BT, such as a high disease burden, chronic course, or suicidal tendencies.

*“I think it is difficult for example with chronical depression or the latest of many recurrent depressive episodes. Especially because of the short time.”* (T008).

### 3.2 Facilitators

**Fig. 2:**
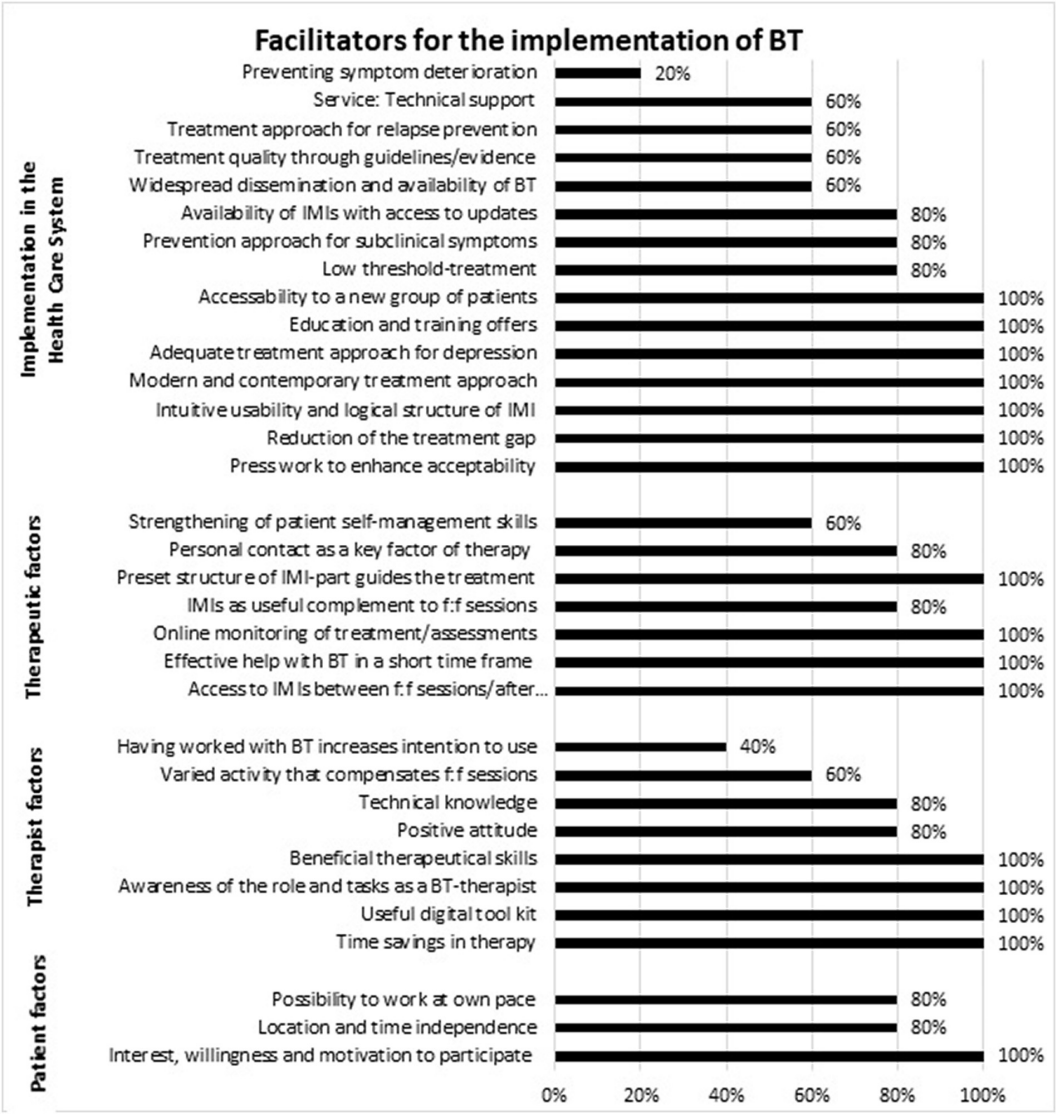
Facilitators per main area and the percentage of therapists mentioning it.

Therapists perceived more facilitating then hindering factors in the 13-weeks BT for therapist factors and implementation factors, and more hindering factors on the level of patients and implementation in the health care system. 48% (*n* = 16) of the identified 33 facilitators were stated by all therapists, 76% (*n* = 25) by at least four. Below, key facilitators that were mentioned by all i the therapists are described with a quotation illustrating the therapists' view. All categories and subthemes and the number of therapists mentioning the respective facilitator are listed in Fig. 2.

#### 3.2.1 Implementation in the health care system

All therapists judged BT as a modern, contemporary and adequate treatment approach, since nearly everyone is familiar with using the internet and has daily access to it. The content of the modules was assessed as therapeutically meaningful as well as relevant for MDD patients, as a therapist expressed below:

*“So what really appealed to me the most was this very nicely designed website with the really well-prepared lessons, which provided information in a nice way and gave examples and exercises.” (T011).*

Other major facilitators stated by all the therapists were that BT can require fewer resources as compared to traditional psychotherapy, and that allows therapists to treat more patients over the same period of time. They indicated also that BT offers the possibility to provide more immediate access to treatment and thereby eventually reduces the treatment gap in mental health care.

*“In the end it is possible to provide more therapy. Since BT is also something young therapists and therapists in training can easily work with, certainly more therapies could be provided.” (T011).*

Therapists judged blended care as an attractive approach for hard to reach patient groups, e.g. men, young patients, people with reservations concerning traditional psychotherapy who might favor using IMIs, and those who might prefer fewer f2f sessions due to conflicts, for example, with other obligations in their daily lives.

All therapists stated the necessity of offering educational and training sessions such as workshops and test-accounts in order to familiarize therapists with the BT approach. In addition, promotional activities could help to enhance the prominence and acceptability of BT. The national press could be enlisted to disseminate information about BT; and investments in advertising and educational activities could be undertaken on a national level.

*“GP's or medical specialists need to have the blended care approach in mind to be able to propose and recommend it to patients.” (T008).*

Furthermore, therapists also appreciated the intuitive usability, design, and logical structure of the online platform and mentioned usability aspects as crucial for implementation targets.

#### 3.2.2 Therapeutic factors

As one important therapeutic factor, all therapists indicated that the online components facilitated the preparation of f2f sessions for both therapists and patients. Patients having access to information between f2f sessions and after finishing treatment was seen as a big advantage, as this facilitated the therapeutic process and allowed patients to repeat lessons on their own and to reflect on their behaviour without having a therapist present. But therapists also liked the possibility of monitoring the treatment course and assessments online and referring to it in f2f sessions. They also noted that the online treatment modules helped them to structure the treatment, which might be beneficial for the overall treatment outcome.

*“Patients can revise what we talked about, get access to all exercises and add something later on. Furthermore, they can repeat some modules after the official end of therapy.” (T007).*

In addition, therapists expressed, that this blended 13-weeks intervention effectively helped to reduce symptoms of depression in a short time frame, as evident in the following sentence:

*“As patients came into treatment everything seemed quite bad, but there was this huge benefit of BT in such a short time.” (T011).*

#### 3.2.3 Therapist factors

All the therapists noted that their time was saved due to the availability of therapy material and pre-gathered information online. Having access to a digital tool kit supported their therapeutic work, as they used a pool of information and exercises with a lot of input to focus on specific issues.

*„It is helpful to know that you have this repertoire of exercises and you can focus on one or the other with every client. Maybe there are some modules that are less useful for some of them and that is also okay.” (T010).*

All therapists felt enabled to perform BT by being aware of the role and tasks of a BT therapist. They reported that they felt no identity conflict with their role, and all were confident that their knowledge of BT was sufficient. They were especially confident that they could integrate the web- and app-based parts into f2f sessions. The therapists observed, as well, that being knowledgeable about the functionalities of the website were important facilitators.

The therapists expressed the following personal traits as essential facilitators for a BT therapist: an empathetic attitude; a high tolerance for frustration; persistency; good writing skills; and the ability to build up a therapeutic alliance with one's patient in a short time frame. Beneficial therapeutic skills were outlined as exemplified below:

*“As a therapist, I also think you should have a certain enthusiasm about online treatments. Not only as a skill, but as a requirement. I need to have some kind of ability to teach and motivate people, why it's worth completing an online lesson additionally.”(T007).*

#### 3.2.4 Patient factors

The therapists indicated that most of their patients expressed great interest and willingness to participate in BT. The therapists observed that their patients felt motivated by this innovative and exciting approach and wanted to engage more in some treatment interventions, such as mood and activities ratings on the app, and using the online calendar in the online portal, in comparison to stand-alone f2f therapy.

*“NONE [patient] would have EVER drawn so many mood curves with a pen. But there is a high intensity of drawing those in the app. I think this is useful even after the end of the project [because they continue with mood ratings].” (T007).*

### 3.3 Identified barriers and facilitators of TDF domains

With reference to the TDF three domains that influence behaviour were identified as barriers, and eleven domains as facilitators. Concerning facilitators, therapists reported they had sufficient knowledge and skills to perform blended care, experienced no identity conflict, and judged blending online modules with f2f therapy as part of their role. They were optimistic according to a positive course and outcome, expressed a high intention and motivation to use it, and found it easy to draw attention to it. Furthermore, therapists expected a positive social influence and felt supported by their positive emotions and behavioral regulation mechanisms while performing BT. Regarding barriers, therapists reported rather reduced self-efficacy because applying BT was also perceived as stressful. Furthermore, they mentioned some hindering environmental context factors (e.g. no existing reimbursement concept) for implementing BT. They also reported their uncertainty about the cost–benefit ratio of BT.

## 4 Discussion

The current study identified important facilitators and barriers for the use and implementation of a 13-weeks BT for depression from therapists' perspective.

The barriers that have been identified by most therapists as crucial for a successful implementation in the health care system were similar to the barriers in previous studies on non-blended, guided stand-alone IMIs and, in general, other e-health solutions; namely, concerns about data safety (Merkel, 2017) and the absence of a clear concept as to how BT can be embedded in the health care system (Drozd et al., 2016; Kivi et al., 2015; Andersson et al., 2009; Alkhaldi et al., 2014; Andersson and Hedman, 2013). Especially important is the lack of funding solutions for the additional effort associated with a blended approach; namely, monitoring online activities, sending emails to remind and motivate their patients to complete the online modules, writing feedback messages to exercises or providing technical support. A partial solution for the necessary additional efforts noted by the therapists (aside from paying the therapist for all of his/her extra work) might be the inclusion of other professionals, such as mental health nurses and/or psychologists, to do the tasks that don't require therapeutic expertise. All therapists identified the repeatedly occurrence of technical problems as barrier to use BT. These technical problems resulted in anger and frustration and distracted the therapy process. Technical problems as hindering factor or the need of user-friendly technical solutions has been repeatedly reported in the literature generated from studies of the use of IMIs for the treatment of depression, e-health solutions in general, and non-blended IMIs (Kivi et al., 2015; Andersson et al., 2009; Alkhaldi et al., 2014; Merkel, 2017). The successful implementation of BT will certainly require a sophisticated technical solution that is free of typical teething problems.

On the other hand the implementation of BT could be facilitated by other factors that have been also found in studies on stand-alone IMIs, e.g. that IMIs were widely accessible to patients and could help to overcome the treatment gap in the health care system (Topooco et al., 2017). If the 13-weeks BT is conceptualized to save therapists time, more patients could potentially be treated with the same number of trained clinicians. However, a recent study showed that offering therapist the option to use online modules to deliver some aspects of treatment online, does not automatically lead to lower costs, as therapists simply provided the online treatment modules on top of the f2f sessions (Kenter et al., 2015). Such a procedure would not reduce the treatment gap. Hence, future research needs to investigate strategies that would motivate therapists to reduce the number of f2f sessions per patient because reducing clinician time per patient is a goal of the 13-weeks BT in Germany. Other important facilitators for the implementation of BT included the offering of training to therapists to acquaint them with the new treatment format (Whitfield and Williams, 2004; Drozd et al., 2016; Perle et al., 2013) and the focusing on dissemination activities designed to enhance the acceptability of BT (Alkhaldi et al., 2014) among therapists and patients. Results showed that several barriers and facilitators for BT are in line with results of previous studies of IMIs and e-Health solutions. If all technology-based solutions for health care would develop together an implementation strategy, profitable synergy effects could emerge.

Furthermore, the use of BT will also be influenced by its possible consequences on the treatment process. The therapists identified several factors that would hinder their therapeutic work: all therapists demanded more autonomy regarding their decisions about how to use BT and more customizability of the online modules. They rejected the ‘one size fits all’ approach. They objected to the blended approach's lack of individualisation. The therapists want the authority to determine the number and ratio of f2f and online sessions for each of their patients. These therapists' objections are in line with the results of a recent Delphi study in which therapists were consulted about the optimal format of blended treatments for depression (van der Vaart et al., 2014) as well as a mixed-method approach with therapists about barriers for implementation of guided IMIs for depression (Kivi et al., 2015). Regarding the therapeutic alliance that is burdened by technical problems, future implementation plans should be wary of providing the technical introduction in the first f2f session. Further, an IT support is needed, independent of the therapists, to address any permanent technical problems that might occur in the IMIs. The therapists also demanded the right to choose and to decide autonomously which of their patients to treat with BT. All the therapists noted several disease-related contraindications, such as severe depression, for instance, that were deemed unsuitable for such a treatment. In line with this finding, the reported acceptability of IMIs for depression was also low in a recent study, if there were contraindications (Topooco et al., 2017). Thus, it might be important to consider BT is not suitable for specific patient groups and to inform therapists systematically about the evidence on moderating factors. Providing case reports, especially for patient groups for which the evidence is available, could be helpful in informing therapists about indications and examples of treatment. The therapists also mentioned that they need additional online content that allow for the treatment of comorbid mental health disorders. This was also recommended in a previous study regarding the implementation of stand-alone IMIs (Andersson et al., 2009).

On the other hand, there are important facilitators regarding the therapy, e.g. most therapists appreciated the online modules as a useful complement of psychotherapy. Thus, they prefer a blended approach, if the f2f sessions are a mandatory and integral portion of BT, while the online modules are complementary. This was in line with a recent study that showed primary care therapists wished to integrate and blend IMIs for depression and f2f therapies because they judged a blended approach as more efficient (Kivi et al., 2015). Another study found that BT was more accepted than stand-alone IMIs, because the lack of personal contact between patient and therapist was seen as a crucial barrier in the implementation of stand-alone IMIs to treat depression (Topooco et al., 2017). In the current study all the therapists also reported some advantages for patients; e.g. that the use of online modules could lead to empowered self-management skills and self-efficacy, which has also been reported as one of the key advantages of BT in one of the few qualitative studies on the therapists' perspective on BT (van der Vaart et al., 2014). This qualitative pilot study yielded new insights from the therapists as to how the blending of f2f and online sessions can facilitate the treatment. These insights were not reported in previous studies. The therapy profits from: (a) patients' access to information between sessions and after the end of treatment modules, which leads to (b) time savings in the f2f sessions, thus enabling therapists to use the additional time to intensify the therapy; (c) the therapists' access to a digital therapy tool kit; (d) therapists' monitoring the online treatment course and use of the assessments, e.g. daily mood ratings, to optimize therapy; and (e) the preset structure of the online platform that guides the treatment course for both the therapists and the patients; while (f) providing to patients a contemporary and modern concept of treatment that fits the patients' needs.

Interestingly, the interviewed therapists reported to have a positive attitude towards this innovative care form, but expected their colleagues to be skeptical of this new approach. Previous studies identified negative attitudes towards e-health solutions in general as barriers (Alkhaldi et al., 2014; Merkel, 2017). But there is also evidence that therapists have an interest and a positive attitude towards the implementation of IMIs in general (Perle et al., 2013) and BT (van der Vaart et al., 2014) in particular, and that is considered to be a facilitator. The therapists in this study mentioned that, after working with BT, they were ready and willing to use it in the future. This seems to be in line with the findings of previous studies where therapists' lack of experience was noted as a hindering factor to the implementation of IMIs for depression (Drozd et al., 2016). So, enabling therapists to experience BT through workshops, through its inclusion in the curriculum of universities and/or training institutes, and through providing free educational test accounts to therapists could promote its usage. While more exposure to BT could potentially reduce the therapists' misperceptions and reservations, probably therapists would be receptive of BT if they understood that their patients are interested in this innovative treatment and are willing and motivated to participate in BT.

The current study has some limitations. The sample consisted of only five therapists, all female. Furthermore, they worked within a structured, standardized treatment approach in the context of a randomized controlled study. In addition, the interviewed therapists had treated a limited number of patients, their average experience differed a lot and experienced therapists might express other views. Hence, generalizability and representativeness cannot be assumed (Mayring, 2002). Furthermore, a wide variability of technical problems might have influenced the therapists' views. Moreover, the interviewed therapists applied for the position to treat patients within a BT approach; hence, results might be biased towards a more positive attitude of BT.

The study's strengths included (a) a theory-based semi-structured interview guide; (b) data collection and analysis by different persons; (c) continuous consensus approach and development of a code list following guidelines of qualitative research (Mayring, 2002); (d) independent coding and high interrater reliability; and e) validation of results by interviewed therapists. Certainly future studies need to explore barriers and facilitators using qualitative and quantitative measurements in larger samples, male therapists or other health care professionals, and therapists that have used BT in the context of routine care, without restrictions with regard to standardization of a randomized trial.

## 5 Conclusion

BT is a new care form in Germany and most therapists are only experienced with performing f2f therapy and not blending it with online sessions in 13 weeks. Therefore, implementation approaches have to consider a wide range of determinants that are crucial for behavior change of therapists. In general, the transfer of evidence into routine practice is often not achieved (Grimshaw et al., 2012; Haines et al., 2004; Glasgow et al., 2003), but behaviour change of therapists could be facilitated by an understanding of influencing barriers and facilitators and using it for a successful implementation (Fixsen et al., 2005). This qualitative study has shown possible facilitators and barriers for the use and implementation of BT for depression from the perspective of five therapists from which implementation efforts can learn. They don't claim to be representative, rather case-descriptive, but deliver in-depth insights and enable possible recommendations to overcome barriers for BT implementation. In addition, future research is needed on some aspects of what therapists suggested, e.g. the flexibility of use of f2f and online modules.

Summarizing our in-depth insights in perceived barriers and facilitators by therapists, we would redesign the BT process for the German health care system in the following manner: Therapists should be able to individualize the treatment and can autonomously decide about the ratio and number of used online modules and f2f sessions. They could choose which patients they treat with BT and could consider disease-related contraindications in their decision. They should have access to online content, e.g. a digital therapy tool kit for comorbid mental health disorders in order to tailor their treatment plan. To reduce the burden of technical issues to the therapeutic alliance, the technical introduction session will follow the first f2f contact and IT support will be provided by IT specialists. The aspects of the online platform could be improved to enhance the usability (e.g. smartphone-based calendar). To reduce time pressure and additional organizational effort, e.g. for monitoring online activities, some of the therapeutic tasks could be delegated. Regarding the future implementation of BT there is a need to develop and provide trainings for therapists. In addition, a concept of embedding BT in the health care system and funding the additional effort has to be developed by involving relevant stakeholders (e.g. health insurances, professional associations, Federal Joint Committee). To prevent frustration and difficulties for patients and therapists when using the online platform, a sophisticated technical solution has to be implemented through cooperation with IT experts. In general, BT could be offered for the treatment of depression and comorbid disorders, but also for relapse prevention.

Another interesting approach to guide implementation efforts is the ‚Fit for Blended Care’ instrument (Wentzel et al., 2016), which aims to facilitate the setting up of a personalized blended treatment by the use of a checklist for barriers and facilitators. All findings from previous, current, or future studies could be summarized and adapted to country-specific settings and be presented as a tool kit to enable professionals to apply BT. The EU project ImpleMentAll (www.implementall.eu) is a current example for such a research approach that aims to provide evidence-based answers to the problems of implementation through the development, application, and evaluation of tailored implementation strategies (ImpleMentAll-Consortium, 2017).

The current study showed that a theory approach with the TDF is also useful for the intervention context of BT applied by therapists. Questions informed by TDF generated 29 barriers and 33 facilitators for what we hypothetically assume to be determinants of behavior change. Each TDF domain was classified as being perceived as a barrier or a facilitator by the therapists. We are now able to use the COM-B model in future implementation efforts and studies. The COM-B model is linked to the TDF and postulates that behavior can be influenced by the components capability, opportunity, and motivation (Michie et al., 2014; Michie et al., 2011). The guide ‘Behavior change wheel’ will provide recommendations about how to use our TDF results and the COM-B model to design interventions and behavior change. In sum, the therapists provided support for the implementation of BT for depression in routine primary care. These five therapists advocated blending online sessions with f2f therapy as an innovative care form and saw the possibility of combining the best of both in their daily work.

The following is the supplementary data related to this article.

Supplementary Table 1 Consolidated criteria for reporting qualitative studies (COREQ): 32-item checklist Supplementary Table 1

## Conflicts of interest

None.

## Acknowledgments and funding

The current study was funded by the European Commission FP7-Health-2013-Innovation-1 program (grant agreement number: 603098) and was conducted by the German trial partner of the project European Comparative Effectiveness Research on Internetbased Depression Treatment (E-COMPARED). The funder had no role in research idea, study design, data collection, analysis and interpretation, decision to publish, or preparation of the manuscript. We thank the therapists and the second coder Steffen Hartmann (SH) for their contributions. Results from the E-COMPARED project can be found at www.e-compared.eu. We acknowledge support by Deutsche Forschungsgemeinschaft and Friedrich-Alexander-Universität Erlangen-Nürnberg (FAU) within the funding programme Open Access Publishing.

## Authors' contributions

IT designed the study, developed the interview guide, collected the data and supervised/contributed to the data analysis and interpretation as well as drafted the manuscript. KS executed data transcription, performed data analysis and interpretation with a second independent coder and contributed to the draft of methods and results. DE supervised the primary stage of developing the study design and the further writing of the manuscript draft, MB and HR critically revised the manuscript.

## References

[bb0005] Alexander C., Fraser J. (2008). General practitioners' management of patients with mental health conditions: the views of general practitioners working in rural north-western New South Wales. Aust. J. Rural Health.

[bb0010] Alkhaldi B., Sahama T., Huxley C., Gajanayake R. (2014). Barriers to implementing eHealth: a multi-dimensional perspective. Studies in Health Technology and Informatics.

[bb0015] Alonso J., Angermeyer M.C., Bernert S. (2004). Prevalence of mental disorders in Europe: results from the European study of the epidemiology of mental disorders (ESEMeD) project. Acta Psychiatr. Scand. Suppl..

[bb0020] Amemori M., Michie S., Korhonen T., Murtomaa H., Kinnunen T.H. (2011). Assessing implementation difficulties in tobacco use prevention and cessation counselling among dental providers. Implement. Sci..

[bb0025] Andersson G., Hedman E. (2013). Effectiveness of guided internet-based cognitive behavior therapy in regular clinical settings. Verhaltenstherapie.

[bb0030] Andersson G., Carlbring P., Berger T. (2009). What Makes Internet Therapy Work?.

[bb0035] Andersson G., Cuijpers P., Carlbring P., Riper H., Hedman E. (2014). Guided internet-based vs. face-to-face cognitive behavior therapy for psychiatric and somatic disorders: a systematic review and meta-analysis guided internet-based vs. face-to-face cognitive behavior therapy for psychiatric and somatic disorders. World Psychiatry.

[bb0040] Andersson G., Topooco N., Havik O., Nordgreen T. (2016). Internet-supported versus face-to-face cognitive behavior therapy for depression. Expert. Rev. Neurother..

[bb0045] Apolinário-Hagen J., Vehreschild V., Alkoudmani R.M. (2017). Current views and perspectives on E-mental health: an exploratory survey study for understanding public attitudes toward internet-based psychotherapy in Germany. J. Med. Internet Res..

[bb0050] Baumeister H., Reichler L., Munzinger M., Lin J. (2014). The impact of guidance on internet-based mental health interventions - a systematic review. Internet Interv..

[bb0055] Baumeister H., Seifferth H., Lin J., Nowoczin L., Lüking M., Ebert D.D. (2014). Impact of an acceptance facilitating intervention on patients' acceptance of internet-based pain interventions - a randomised controlled trial. Clin. J. Pain.

[bb0060] Baumeister H., Nowoczin L., Lin J. (2014). Impact of an acceptance facilitating intervention on diabetes patients' acceptance of internet-based interventions for depression: a randomized controlled trial. Diabetes Res. Clin. Pract..

[bb0065] Baumeister H., Lin J., Ebert D.D. (2017). Internet-und mobilebasierte Ansätze. Bundesgesundheitsbl. Gesundheitsforsch. Gesundheitsschutz.

[bb0070] Berger T., Krieger T., Sude K., Meyer B., Maercker A. (2017). Evaluating an e-mental health program (“deprexis”) as adjunctive treatment tool in psychotherapy for depression: results of a pragmatic randomized controlled trial. J. Affect. Disord..

[bb0075] Brown R.D., Hauenstein N.M.A. (2005). Interrater agreement reconsidered: an alternative to the rwg indices. Organ. Res. Methods.

[bb0080] Brownson R.C., Colditz G.A., Proctor E.K. (2012). Dissemination and Implementation Research in Health: Translating Science to Practice.

[bb0085] Buti A.L., Eakins D., Fussell H., Kunkel L.E., Kudura A., McCarty D. (2013). Clinician attitudes, social norms and intentions to use a computer-assisted intervention. J. Subst. Abus. Treat..

[bb0090] Campbell M. (2000). Framework for design and evaluation of complex interventions to improve health. BMJ.

[bb0095] Campbell N.C., Murray E., Darbyshire J. (2007). Designing and evaluating complex interventions to improve health care. BMJ.

[bb0100] Cane J., O'Connor D., Michie S. (2012). Validation of the theoretical domains framework for use in behaviour change and implementation research. Implement. Sci..

[bb0105] Cuijpers P., Van Straten A., Andersson G. (2008). Internet-administered cognitive behavior therapy for health problems: a systematic review. J. Behav. Med..

[bb0110] Cuijpers P., Cristea I.A., Karyotaki E., Reijnders M., Huibers M.J.H. (2016). How effective are cognitive behavior therapies for major depression and anxiety disorders? A meta-analytic update of the evidence. World Psychiat..

[bb0115] Cunningham J.A., Khadjesari Z., Bewick B.M., Riper H. (2010). Internet-based interventions for problem drinkers: from efficacy trials to implementation. Drug Alcohol Rev..

[bb0120] Drozd F., Vaskinn L., Bergsund H.B., Haga S.M., Slinning K., Bjørkli C.A. (2016). The implementation of internet interventions for depression: a scoping review. J. Med. Internet Res..

[bb0125] Ebert D.D., Berking M., Cuijpers P., Lehr D., Pörtner M., Baumeister H. (2015). Increasing the acceptance of internet-based mental health interventions in primary care patients with depressive symptoms. A randomized controlled trial. J. Affect. Disord..

[bb0130] Ebert DD, Buntrock C, Lehr D, et al. Effectiveness of web- and mobile-based treatment of subthreshold depression with adherence-focused guidance: a single-blind randomized controlled trial. Behav. Ther.. http://linkinghub.elsevier.com/retrieve/pii/S000578941730059X. (Published) 2017. Accessed July 18, 2017. 10.1016/j.beth.2017.05.004 29405923

[bb0135] Eccles M., Grimshaw J., Walker A., Johnston M., Pitts N. (2005). Changing the behavior of healthcare professionals: the use of theory in promoting the uptake of research findings. J. Clin. Epidemiol..

[bb0140] Erbe D., Eichert H.-C., Riper H., Ebert D.D. (2017). Blending face-to-face and internet-based interventions for the treatment of mental disorders in adults: systematic review. J. Med. Internet Res..

[bb0145] Fixsen D.L., Naoom S.F., Blase K.A., Friedman R.M., Wallace F. (2005). Implementation Research: A Synthesis of the Literature.

[bb0150] French S.D., Green S.E., O'Connor D.A. (2012). Developing theory-informed behaviour change interventions to implement evidence into practice: a systematic approach using the theoretical domains framework. Implement. Sci..

[bb0155] Glasgow R.E., Lichtenstein E., Marcus A.C. (2003). Why don't we see more translation of health promotion research to practice? Rethinking the efficacy-to-effectiveness transition. Am. J. Public Health.

[bb0160] Greenhalgh T., Robert G., Macfarlane F., Bate P., Kyriakidou O. (2004). Diffusion of innovations in service organizations: systematic review and recommendations. Milt. Q..

[bb0165] Grimshaw J.M., Eccles M.P., Lavis J.N., Hill S.J., Squires J.E. (2012). Knowledge translation of research findings. Implement. Sci..

[bb0170] Grol R., Bosch M.C., Hulscher M.E., Eccles M.P., Wensing M. (2007). Planning and studying improvements in patient care: the use of theoretical perspectives. Milt. Q..

[bb0175] Haines A., Kuruvilla S., Borchert M. (2004). Bridging the implementation gap between knowledge and action for health. Bull. World Health Organ..

[bb0180] Huijg J.M., W a Gebhardt, Crone M.R., Dusseldorp E., Presseau J. (2014). Discriminant content validity of a theoretical domains framework questionnaire for use in implementation research. Implement. Sci..

[bb0185] ImpleMentAll-Consortium (2017). Home - ImpleMentAll. http://www.implementall.eu/.

[bb0190] Karyotaki E., Riper H., Twisk J. (2017). Efficacy of self-guided internet-based cognitive behavioral therapy in the treatment of depressive symptoms. JAMA Psychiatry.

[bb0195] Kazdin A.E., Blase S.L. (2011). Rebooting psychotherapy research and practice to reduce the burden of mental illness. Perspect. Psychol. Sci..

[bb0200] Kemmeren L.L., van Schaik D.J.F., Riper H., Kleiboer A.M., Bosmans J.E., Smit J.H. (2016). Effectiveness of blended depression treatment for adults in specialised mental healthcare: study protocol for a randomised controlled trial. BMC Psychiatry..

[bb0205] Kenter R.M.F., van de Ven P.M., Cuijpers P. (2015). Costs and effects of internet cognitive behavioral treatment blended with face-to-face treatment: results from a naturalistic study. Internet Interv..

[bb0210] Kessler R.C., Berglund P.A., Bruce M.L. (2001). The prevalence and correlates of untreated serious mental illness. Health Serv. Res..

[bb0215] Kivi M., Eriksson M.C.M., Hange D., Petersson E.L., Björkelund C., Johansson B. (2015). Experiences and attitudes of primary care therapists in the implementation and use of internet-based treatment in Swedish primary care settings. Internet Interv..

[bb0220] Kleiboer A., Smit J., Bosmans J. (2016). European COMPARative effectiveness research on blended depression treatment versus treatment-as-usual (E-COMPARED): study protocol for a randomized controlled, non-inferiority trial in eight European countries. Trials.

[bb0225] Klein J.P., Gerlinger G., Knaevelsrud C. (2016). Internetbasierte Interventionen in der Behandlung psychischer Störungen: Überblick, Qualitätskriterien, Perspektiven. Nervenarzt.

[bb0230] Königbauer J., Letsch J., Doebler P., Ebert D., Baumeister H. (2017). Internet- and mobile-based depression interventions for people with diagnosed depression: a systematic review and meta-analysis. J. Affect. Disord..

[bb0235] Kooistra L.C., Ruwaard J., Wiersma J.E. (2016). Development and initial evaluation of blended cognitive behavioural treatment for major depression in routine specialized mental health care. Internet Interv..

[bb0240] LA McSherry, Dombrowski S.U., Francis J.J. (2012). “It”s a can of worms': understanding primary care practitioners' behaviours in relation to HPV using the theoretical domains framework. Implement. Sci..

[bb0245] Ly K.H., Topooco N., Cederlund H. (2015). Smartphone-supported versus full behavioural activation for depression: a randomised controlled trial. PLoS One.

[bb0250] Mack S., Jacobi F., Gerschler A. (2014). Self-reported utilization of mental health services in the adult german population - evidence for unmet needs? Results of the DEGS1-mental health module (DEGS1-MH). Int. J. Methods Psychiatr. Res..

[bb0255] May C., Finch T. (2009). Implementing, embedding, and integrating practices: an outline of normalization process theory. Sociology.

[bb0260] Mayring P. (2002). Einführung in Die Qualitative Sozialforschung: Eine Anleitung Zu Qualitativem Denken (5., Überarb. Und Neu Ausgestattete Aufl.). Studium Paedagogik.

[bb0265] Mayring P. (2010). Qualitative Inhaltsanalyse: Grundlagen Und Techniken (11., Aktualisierte Auflage Und Überarb. Aufl.). Studium Paedagogik.

[bb0270] Merkel S. (2017). Umsetzungsbarrieren bei der Akzeptanz, Implementation und Verbreitung von Telecare und Telehealth - Ergebnisse einer internationalen Literaturstudie. E-Health-Ökonomie.

[bb0275] Michie S., Johnston M., Francis J., Hardeman W., Eccles M. (2008). From theory to intervention: mapping theoretically derived behavioural determinants to behaviour change techniques. Appl. Psychol..

[bb0280] Michie S., van Stralen M.M., West R. (2011). The behaviour change wheel: a new method for characterising and designing behaviour change interventions. Implement. Sci..

[bb0285] Michie S., Atkins L., West R. (2014). The Behaviour Change Wheel: A Guide to Designing Interventions.

[bb0290] Murray E., Treweek S., Pope C. (2010). Normalisation process theory: a framework for developing, evaluating and implementing complex interventions. BMC Med..

[bb0295] Musiat P., Goldstone P., Understanding Tarrier N. (2014). The acceptability of e-mental health - attitudes and expectations towards computerised self-help treatments for mental health problems. BMC Psychiatry.

[bb0300] Nilsen P. (2015). Making sense of implementation theories, models and frameworks. Implement. Sci..

[bb0305] Patey A.M., Islam R., Francis J.J., Bryson G.L., Grimshaw J.M. (2012). Anesthesiologists' and surgeons' perceptions about routine pre-operative testing in low-risk patients: application of the theoretical domains framework (TDF) to identify factors that influence physicians' decisions to order pre-operative tests. Implement. Sci..

[bb0310] Perle J.G., Langsam L.C., Randel A. (2013). Attitudes toward psychological telehealth: current and future clinical psychologists. Opin. Internet-Based Interv..

[bb0315] Piek E., van der Meer K., Penninx B.W.J.H., Verhaak P.F.M., Nolen W.A. (2011). Referral of patients with depression to mental health care by Dutch general practitioners: an observational study. BMC Fam. Pract..

[bb0320] Richards D., Richardson T. (2012). Computer-based psychological treatments for depression: a systematic review and meta-analysis. Clin. Psychol. Rev..

[bb0325] Richards J.C., Ryan P., McCabe M.P., Groom G., Hickie I.B. (2004). Barriers to the effective management of depression in general practice. Aust. N. Z. J. Psychiatry.

[bb0330] Sander L., Ebert D., Baumeister H. (2017). Internet- und mobilebasierte Psychotherapie der Depression. Fortschr. Neurol. Psychiatr..

[bb0335] Taylor N., Parveen S., Robins V., Slater B., Lawton R. (2013). Development and initial validation of the influences on patient safety behaviours questionnaire. Implement. Sci..

[bb0340] Topooco N., Riper H., Araya R. (2017). Attitudes towards digital treatment for depression: a European stakeholder survey. Internet Interv..

[bb0345] van der Vaart R., Witting M., Riper H., Kooistra L., Bohlmeijer E.T., van Gemert-Pijnen L.J. (2014). Blending online therapy into regular face-to-face therapy for depression: content, ratio and preconditions according to patients and therapists using a Delphi study. BMC Psychiatry.

[bb0350] Wang J., Langille D.B., Patten S.B. (2003). Mental health services received by depressed persons who visited general practitioners and family doctors. Psychiatr. Serv..

[bb0355] Wangberg S.C., Gammon D., Spitznogle K. (2007). In the Eyes of the Beholder: Exploring Psychologists' Attitudes Towards and Use of e-Therapy in Norway.

[bb0360] Wentzel J., van der Vaart R., Bohlmeijer E.T., van Gemert-Pijnen J.E.W.C. (2016). Mixing online and face-to-face therapy: how to benefit from blended care in mental health care. J. Med. Internet Res..

[bb0365] Whitfield G., Williams C. (2004). If the evidence is so good – why doesn't anyone use them? A national survey of the use of computerized cognitive behaviour therapy. Behav. Cogn. Psychother..

