# Supplementary material for: Barriers and facilitators for the implementation of blended psychotherapy for depression: A qualitative pilot study of therapists' perspective
Source: Internet Interv. 2018 Jan 16;12:150–64. doi: 10.1016/j.invent.2018.01.002 (PMC6096333; doi:10.1016/j.invent.2018.01.002)
Supplement: Supplementary Table 1 — Consolidated criteria for reporting qualitative studies (COREQ): 32-item checklist [file mmc1.docx]

| **Supplementary Table 1**  Consolidated criteria for reporting qualitative studies (COREQ): 32-item checklist | | |
| --- | --- | --- |
| **No. Item** | **Guide questions/description** | **Reported** |
| **Domain 1: Research team and reﬂexivity** | | |
| *Personal Characteristics* | | |
| 1. Interviewer/ facilitator | Which author/s conducted the interview or focus group? | Titzler, Ingrid, M.Sc. |
| 2. Credentials | What were the researcher’s credentials? E.g. PhD, MD | Master of Science  PhD Student |
| 3. Occupation | What was their occupation at the time of the study? | Trial coordinator *E-Compared,* scientific research staff |
| 4. Gender | Was the researcher male or female? | Female |
| 5. Experience and training | What experience or training did the researcher have? | 3 years research experience in health care and treatment; workshop for qualitative studies; exchange experiences with qualitative researcher; experience as clinical therapist, professional guidance of blended therapists |
| *Relationship with participants* | | |
| 6. Relationship established | Was a relationship established prior to study commencement? | Yes. Participants were well known through their occupation as a therapist in the E-Compared project. |
| 7. Participant knowledge of the interviewer | What did the participants know about the researcher? e.g. personal goals, reasons for doing the research | The participants knew all tasks and responsibilities of the researcher in the project.  Personal goals: none.  Reasons for doing the expert interviews were known and stated at beginning of the interviews. |
| 8. Interviewer characteristics | What characteristics were reported about the interviewer/facilitator? e.g. Bias, assumptions, reasons and interests in the research topic | Participants were told that the interviewer is interested in implementation science and the aim of the interview was getting insights in relevant factors to use them for later implementation projects of blended therapy. |

| **Supplementary Table 1**  COREQ (continued) | | |
| --- | --- | --- |
| **No. Item** | **Guide questions/description** | **Reported** |
| **Domain 2: study design** | | |
| *Theoretical framework* | | |
| 9. Methodological orientation and Theory | What methodological orientation was stated to underpin the study? e.g. grounded theory, discourse analysis, ethnography, phenomenology, content analysis | Content Analysis,  Theory (TDF) basis of interview guide, inductive and deductive content analysis |
| *Participant selection* | | |
| 10. Sampling | How were participants selected? e.g. purposive, convenience, consecutive, snowball | Purposive |
| 11. Method of approach | How were participants approached? e.g. face-to-face, telephone, mail, email | Face-to-face, email |
| 12. Sample size | How many participants were in the study? | 5 took part |
| 13. Non-participation | How many people refused to participate or dropped out? Reasons? | 3 refused to participate because of interference with current occupation; they stopped working in the project before the study was planned |
| *Setting* |  |  |
| 14. Setting of data collection | Where was the data collected? e.g. home, clinic, workplace | Workplace |
| 15. Presence of non-participants | Was anyone else present besides the participants and researchers? | No |
| 16. Description of sample | What are the important characteristics of the sample? e.g. demographic data, date | Young women with a university degree in psychology and in further education as advanced clinical therapist (description made) |
| *Data collection* |  |  |
| 17. Interview guide | Were questions, prompts, guides provided by the authors? Was it pilot tested? | Questions, prompts & guides were provided. Pilot testing with the first interview. Reflection about items with the participant after the first interview. No difficulties to be considered. A similar interview guide successfully used in prior study with general practitioners. |

| **Supplementary Table 1**  COREQ (continued) | | |
| --- | --- | --- |
| **No. Item** | **Guide questions/description** | **Reported** |
| 19. Audio/visual recording | Did the research use audio or visual recording to collect the data? | Audio recording was used |
| 20. Field notes | Were ﬁeld notes made during and/or after the interview or focus group? | Yes, field notes were made during the interview |
| 21. Duration | What was the duration of the interviews or focus group? | *M* = 99 minutes, *SD* = 12.10, *Min* = 86 minutes, *Max* = 112 minutes |
| 23. Transcripts returned | Were transcripts returned to participants for comment and/or correction? | No |
| **Domain 3: analysis and ﬁndings** | | |
| *Data analysis* |  |  |
| 24. Number of data coders | How many data coders coded the data? | 2 |
| 25. Description of the coding tree | Did authors provide a description of the coding tree? | Yes |
| 26. Derivation of themes | Were themes identiﬁed in advance or derived from the data? | Themes identified in advance as part of the theoretical framework and derived from the data as an inductive approach as well |
| 27. Software | What software, if applicable, was used to manage the data? | MAXQDA, Version 12, F4, Audacity |
| 28. Participant checking | Did participants provide feedback on the ﬁndings? | Yes, agreement to identified categories was required |
| *Reporting* |  |  |
| 29. Quotations presented | Were participant quotations presented to illustrate the themes/ﬁndings? Was each quotation identiﬁed (e.g. ID)? | Yes |
| 30. Data and ﬁndings consistent | Was there consistency between the data presented and the ﬁndings? | Yes |
| 31. Clarity of major themes | Were major themes clearly presented in the ﬁndings? | Yes |
| 32. Clarity of minor themes | Is there a description of diverse cases or discussion of minor themes? | Yes |
| Note. Tong A, Sainsbury P, Craig J. Consolidated criteria for reporting qualitative research (COREQ): a 32-item checklist for interviews and focus groups. International Journal for Quality in Health Care. 2007. Volume 19, Number 6: pp. 349 – 357. | | |
